# The recent advances in vaccine adjuvants

**DOI:** 10.3389/fimmu.2025.1557415

**Published:** 2025-05-13

**Authors:** Jiayin Xing, Xiangxiang Zhao, Xiaotian Li, Ren Fang, Mingrui Sun, Yang Zhang, Ningning Song

**Affiliations:** Weifang Key Laboratory of Respiratory Tract Pathogens and Drug Therapy, School of Life Science and Technology, Shandong Second Medical University, Weifang, China

**Keywords:** adjuvants, delivery systems, immunostimulants, vaccine, combinatorial adjuvant strategies

## Abstract

Vaccine adjuvants, as key components in enhancing vaccine immunogenicity, play a vital role in modern vaccinology. This review systematically examines the historical evolution and mechanisms of vaccine adjuvants, with particular emphasis on innovative advancements in aluminum-based adjuvants, emulsion-based adjuvants, and nucleic acid adjuvants (e.g., CpG oligonucleotides). Specifically, aluminum adjuvants enhance immune responses through particle formation/antigen adsorption, inflammatory cascade activation, and T-cell stimulation. Emulsion adjuvants amplify immunogenicity via antigen depot effects and localized inflammation, while nucleic acid adjuvants like CpG oligonucleotides directly activate B cells and dendritic cells to promote Th1-type immune responses and memory T-cell generation. The article further explores the prospective applications of these novel adjuvants in combating emerging pathogens (including influenza and SARS-CoV-2), particularly highlighting their significance in improving vaccine potency and durability. Moreover, this review underscores the critical importance of adjuvant development in next-generation vaccine design and provides theoretical foundations for creating safer, effective adjuvant.

## 1 Introduction

The application of vaccines has significantly reduced the incidence and mortality rates of infectious diseases, highlighting their profound impact on public health. However, traditional vaccines have limitations in their protective efficacy against specific diseases, such as weak immunogenicity and challenges in eliciting robust immune responses, particularly in vaccines for influenza and Human papillomavirus (HPV) (1, 2). The currently approved acellular pertussis vaccines primarily provide protection by inducing antibody responses, but they weakly stimulate cellular immunity and Th1 responses. Furthermore, although the aP vaccine generates a strong antibody response initially, antibody levels decrease over time, leading to a gradual weakening of immune memory (3). To break above these limitations, adjuvants have been developed to enhance the immunogenic effects of vaccines. Effective adjuvants enable to improve vaccine efficacy via several mechanisms: eliciting specific immune responses, prolonging their duration, increasing the avidity and affinity of antibodies produced, stimulating cytotoxic T lymphocyte (CTL) responses, increasing response rates in individuals with lower responsiveness, and benefiting immunocompromised patients (4). Therefore, adjuvants adding into vaccines can stimulate the immune system to enhance the intensity and duration of the immune response (5), thereby broadening the application scope of vaccines.

**Figure 1:**
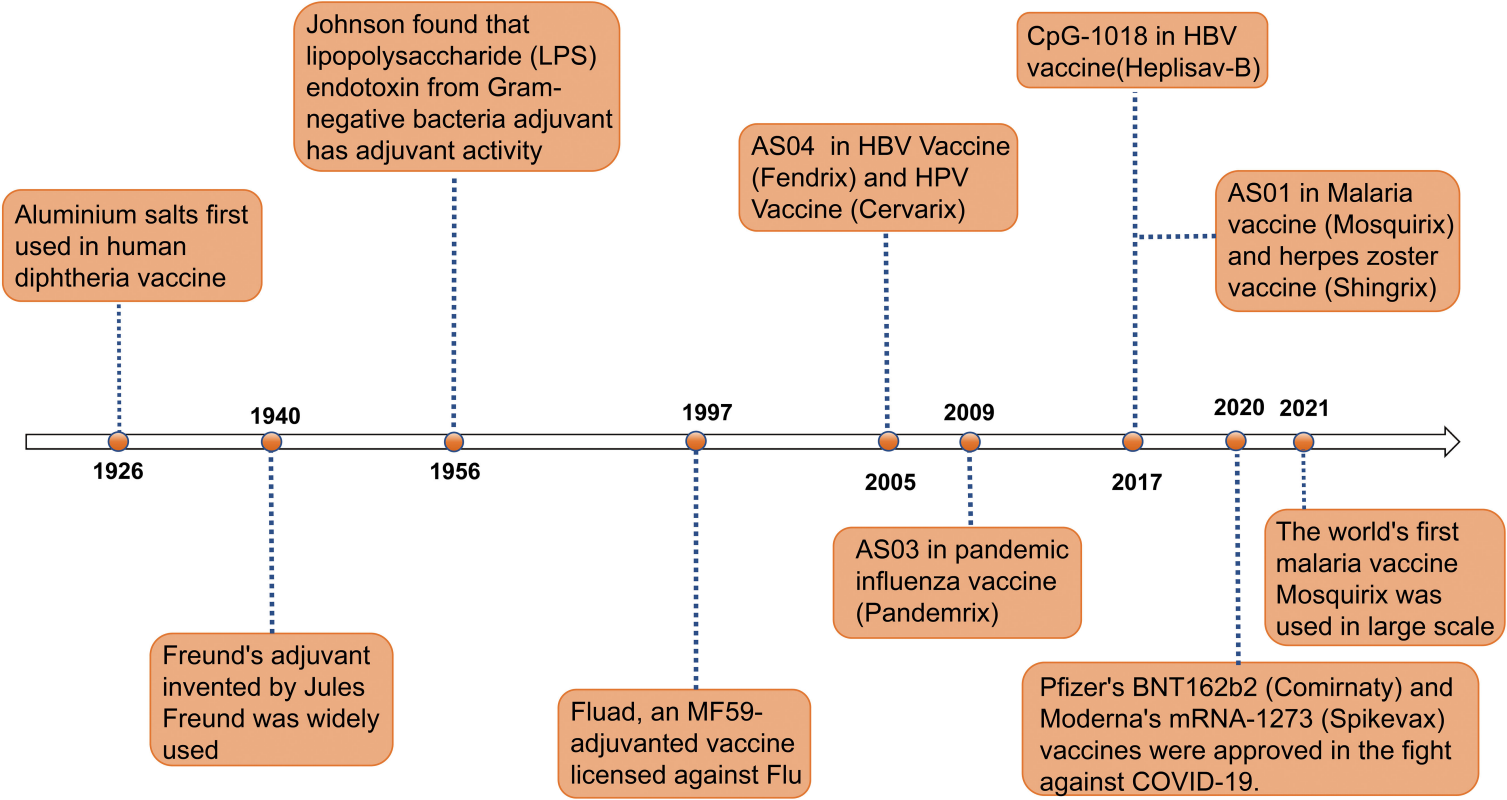
Timeline of major events in the research history of vaccine adjuvants. Since the first use of aluminum salt adjuvants in diphtheria vaccines in 1926, adjuvant technology has gradually evolved (7). In 1940, the invention of Freund’s adjuvant provided a new direction for enhancing immune responses in vaccines (7). In 1956, the discovery of the adjuvant activity of lipopolysaccharide (LPS) endotoxins further expanded the range of available adjuvants. In 1997, the application of the MF59 adjuvant in the Fluad influenza vaccine marked the significant role of adjuvants in influenza prevention and treatment (7). In 2005, the AS04 adjuvant was first used in HBV and HPV vaccines (Fendrix and Cervarix) (8). In 2009, the AS03 adjuvant was used in the pandemic influenza vaccine Pandemrix (9). In 2017, the CpG-1018 and AS01 adjuvants were applied to the HBV vaccine (Heplisav-B) and malaria and shingles vaccines (Mosquirix and Shingrix), respectively (10, 11). In 2020, Pfizer’s BNT162b2 vaccine (Comirnaty) and Moderna’s mRNA-1273 vaccine (Spikevax) were approved, making a significant contribution to the fight against COVID-19. In 2021, the world’s first malaria vaccine RTS,S/AS01 began large-scale use, further proving the role of adjuvants in enhancing vaccine efficacy (12). This timeline illustrates the continuous innovation and breakthroughs of adjuvants in the field of vaccines.

Since 1920s, it has been discovered that aluminum adjuvants could significantly enhance the immune response to diphtheria and tetanus toxoids, marking a pivotal milestone in vaccine development (6) (**Figure 1**). For decades, aluminum adjuvants act as sole adjuvants by slowly releasing antigen from immune sites. However, their effectiveness is limited by lack of diversity and specificity (13). To meet the requirements for various vaccines, some novel adjuvants are developed to apply in approved human vaccines, such as MF59, AS01, AS03, AS04, and CpG 1018 (14) (**Table 1**). The introduction of these new adjuvants has broken the singular paradigm of using aluminum adjuvants as the sole adjuvant, significantly expanding and improving the types and functions of vaccines. Although limited adjuvants are approved for human, their development greatly promote the discover of new functional vaccines (20).

**Table 1:** Classification of adjuvants.

Adjuvant Class		Immune Responses	Licensed vaccines	Ref.
Aluminum adjuvants		promotes antigen uptake by APCs, activates NLRP3 inflammasome, and induces Th2 type immune response.	Hepatitis A vaccine; Hepatitis B vaccine; Diphtheria/tetanus/pertussis (DTP) vaccine.	(15)
Emulsions	MF59	Enhances recruitment of APCs and their activation, promotes antigen uptake and migration of immune cells to lymph nodes, modulates humoral and cellular immune responses.	Seasonal influenza vaccine; Pandemic influenza vaccine; Avian influenza vaccine.	(16)
	AS03	Induces cytokine production and recruitment of immune cells, modulates humoral and cellular immunity.	Pre-pandemic H5N1 vaccine; Pandemic H1N1 influenza vaccine.	(17)
Particles	VLPs	Direct activation of B cells, stimulation of DCs, and induction of cross-presentation to CD8^+^ T cells		(18)
	Virosomes	Promotes uptake of vaccine antigen by APCs and interacts with B cells leading to T-cell activation.		(8)
Mannan-based adjuvants		Promotes DC maturation and skews immune responses toward tolerance or the differentiation of Th1/regulatory T cells (Tregs)		(19)
TLR3 agonists	Poly(I:C)	Enhances antibody titer, Th1 type immunity and CD8^+^ T cell-mediated immunity.		(2)
TLR5 agonist	Flagellin	Enhances Th1 and Th2 immune responses, activates pattern recognition receptors (PRRs), induces strong mucosal IgA/Th2/Th17 responses.		(20)
TLR9 agonist	CpG 1018	Boosts the humoral immune response, Th1 type immunity, CD8^+^ T cell-mediated immunity.	Hepatitis B vaccine (Heplisav-B).	(10)
Combination of adjuvants	AS01	Augments the antibody titer, Th1 type of immune response, and CD8^+^ T cell-mediated immunity	Malaria vaccine (RTS, S or Mosquirix); Herpes zoster vaccine (HZ/su or Shingrix)	(11)
	AS02	Enhances antibody titer and Th1 type immunity.		(2)
	AS04	Stimulates TLR4, increases APC maturation, enhances Th1 type immune responses, and improves humoral and cellular immune responses.	HPV vaccine (Cervarix); Hepatitis B vaccine (Fendrix).	(8)

Vaccine adjuvants come in a wide variety, which can be classified based on criteria such as physicochemical properties, source, type, and mechanism of action, highlighting their importance in vaccine development (21). Based on their action mechanisms, the function of adjuvants can be categorized into delivery systems and immunostimulants (22). The adjuvants act as a delivery system that loads antigens and enhances the uptake and presentation of these antigens by antigen-presenting cells (APCs), primarily functioning to facilitate antigen presentation. The antigen presentation process involves the recognition, uptake, and internalization of antigens by APCs, followed by loading and presenting the antigens on the APC surface via major histocompatibility complex (MHC) molecules (23). The adjuvants act as immunostimulants to promote the maturation and activation of APCs by targeting specific receptors on these cells, thereby enhancing immune responses. Immunostimulants act as pathogen-associated molecular patterns (PAMPs), damage-associated molecular patterns (DAMPs), or their mimics, interacting with pattern recognition receptors (PRRs) on APCs to trigger innate immune responses and lead to APC activation and maturation (7). Mature APCs reduce the phagocytic activity toward antigens and enhance their ability to present antigens, provide co-stimulatory signals, and express cytokines, thereby initiating and amplifying adaptive immune responses (24).

This review categorized adjuvants based on their mechanisms of action, and summarized the distinct mechanisms and immunological characteristics of delivery systems, immunostimulants, and their combinations with classic adjuvants. We also discussed potential future directions for adjuvant development, including innovations in formulation and applications for emerging infectious diseases. This review aimed to provide valuable insights for further investigating the mechanisms of adjuvants and novel adjuvants for enhanced vaccine efficacy.

## 2 Delivery systems

The adjuvant delivery system significantly enhances vaccine immunogenicity by optimizing the efficient delivery of antigens, boosting immune cell activation, controlling antigen release rate, and prolonging the duration of immune responses. Adjuvant delivery platforms have become crucial immune-enhancing strategies in vaccine development, such as aluminum adjuvants, emulsions (e.g., MF59, AS03), and particles (e.g., virus-like particles, virosomes), serving as important tools to enhance immunogenicity.

### 2.1 Aluminum adjuvants

Aluminum adjuvants generally refer to a mixture of compounds such as aluminum hydroxide (Al(OH)_3_) and aluminum phosphate (AlPO_4_), which serve as adjuvants to enhance vaccine efficacy (25). As the most widely used adjuvants in human vaccines, they have been clinically approved (8). In the current clinical trial landscape, vaccines formulated with aluminum adjuvants are being utilized for protection against numerous infectious diseases including COVID-19, and pertussis (15). Aluminum adjuvants and antigens form the complexes through their interaction, thereby facilitating the delivery of antigens to APCs for enhancing uptake.

**Figure 2:**
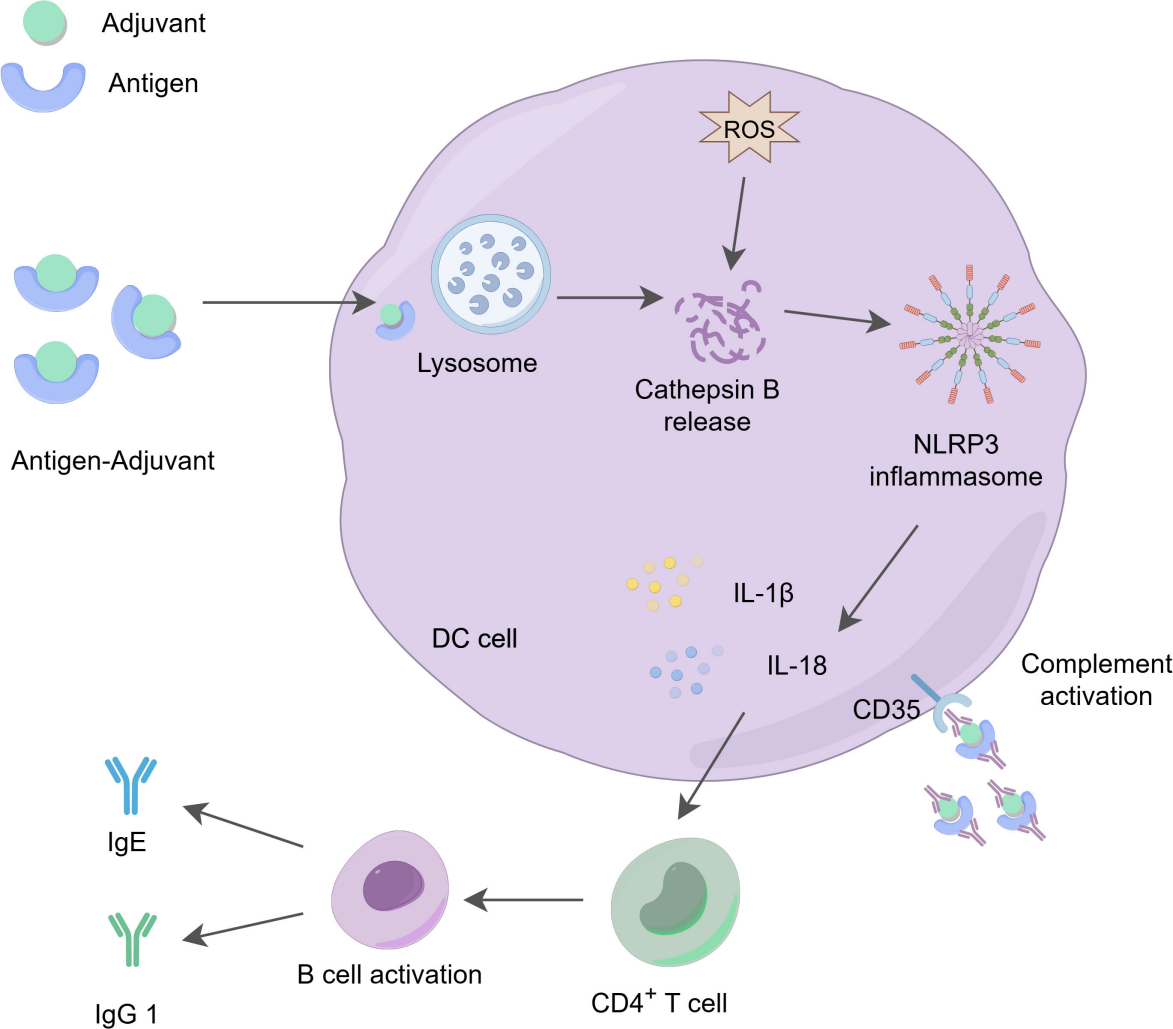
The action mechanism of aluminum adjuvants. Aluminum adjuvants form microparticles by adsorbing soluble antigens, which promotes the phagocytosis of these antigens by APCs. The phagocytosed aluminum-adjuvant-antigen complexes indirectly promote the production of reactive oxygen species (ROS) (13), activate the release of cathepsin B in the lysosome, triggering the activation of the NLRP3 inflammasome and stimulating the production of IL-1β and IL-18, which play a role in regulating immune responses. At the same time, aluminum adjuvants stimulate the activation and differentiation of CD4^+^ T cells, increasing the levels of IgG1 and IgE. These cytokines and immune factors are essential for effective antibody-mediated immune protection. Additionally, dendritic cells can recruit and deposit antigen-adjuvant-antibody complexes through the CD35 receptor (31), further enhancing receptor signaling on both B cells and dendritic cells, thereby promoting immune effector functions.

The action mechanism of aluminum adjuvant is as follows: 1) they directly bind to lipids on the membranes of dendritic cells (DCs), which enhances their ability to present antigens. 2) they form particles through adsorption with soluble antigens, promoting the phagocytic uptake of these antigens by APCs, thereby strengthening the immune response (26, 27). The phagocytosed alum-antigen complexes trigger the release of cathepsin B from lysosomes into the cytoplasm, activating the caspase-1-related NLRP3 inflammasome (28). Subsequently, caspase-1 catalyzes the production of pro-inflammatory cytokines, such as IL-1β, IL-18, and IL-33, which play crucial roles in mediating immune responses (20). Aluminum adjuvants activate several key signaling pathways through its interaction with DCs, including the phosphoinositide 3-kinase pathway and the calcineurin-nuclear factor of activated T cells (NFAT) pathway, which depend on spleen tyrosine kinase (Syk) activity (29, 30) (**Figure 2**). 3) As immunostimulants, they can induce the production of DAMPs, which activate PRRs in the innate immune pathway, leading to the secretion of cytokines such as IL-1β and the initiation of a Th2 immune response (7). Aluminum adjuvants promote humoral immunity by inducing a Th2 immune response, characterized by increased levels of IgG1, IgE, IL-4, IL-5, and eosinophils, which are crucial for effective antibody-mediated protection (32, 33). Therefore, aluminum adjuvants effectively facilitate the generation of high-titer and long-lasting antibody responses, contributing to its overall effectiveness in vaccines.

However, aluminum adjuvants have limitations in effectively inducing robust cellular immune responses and may lead to adverse reactions such as erythema and allergic responses, primarily due to its low immunogenicity and irritability. Therefore, improving the formulation of aluminum adjuvants or developing them as nano-alum adjuvants could enhance their immunogenic effects and address current limitations, potentially leading to more effective vaccines (34, 35).

### 2.2 Emulsion

Emulsions, which are mixtures of two or more liquid phases, have a long history of use as vaccine adjuvants to enhance the immune response to antigens (4). These emulsions are two-phase systems that require surfactants to stabilize the oil-in-water composition. They can be categorized into several types, including water-in-oil (W/O), oil-in-water (O/W), and multiple emulsions (36).

#### 2.2.1 Water-in-oil adjuvant: Freund’s adjuvant

Freund’s adjuvant is a stable water-in-oil emulsion made from paraffin oil and lanolin. Complete Freund’s Adjuvant (CFA) contains inactivated and dried *Mycobacterium tuberculosis*, while incomplete Freund’s adjuvant (IFA) does not (37). Both forms play a crucial role in enhancing immunogenicity (4). CFA acts as an adjuvant by prolonging the lifespan of the injected antigen and facilitating its effective delivery to the immune system (38). However, reports suggest that CFA can induce toxic effects, such as lesions at the injection site, granulomas, and inflammation (4). IFA functions as an adjuvant by forming an oily depot for the antigen, which allows for its continuous release at the injection site. This mechanism not only extends the antigen’s lifespan but also triggers a strong local immune response, including phagocytosis, leukocyte recruitment, infiltration, and cytokine production. Despite its effectiveness, the widespread use of IFA in vaccines is limited due to significant side effects, primarily caused by the toxicity of non-biodegradable oils of poor quality. A 2005 World Health Organization survey showed that among approximately 1 million people vaccinated with IFA-containing vaccines, 40,000 experienced severe side effects, such as aseptic abscesses (22).

#### 2.2.2 Oil-in-water adjuvant: MF59, AS03

##### 2.2.2.1 MF59

**Figure 3:**
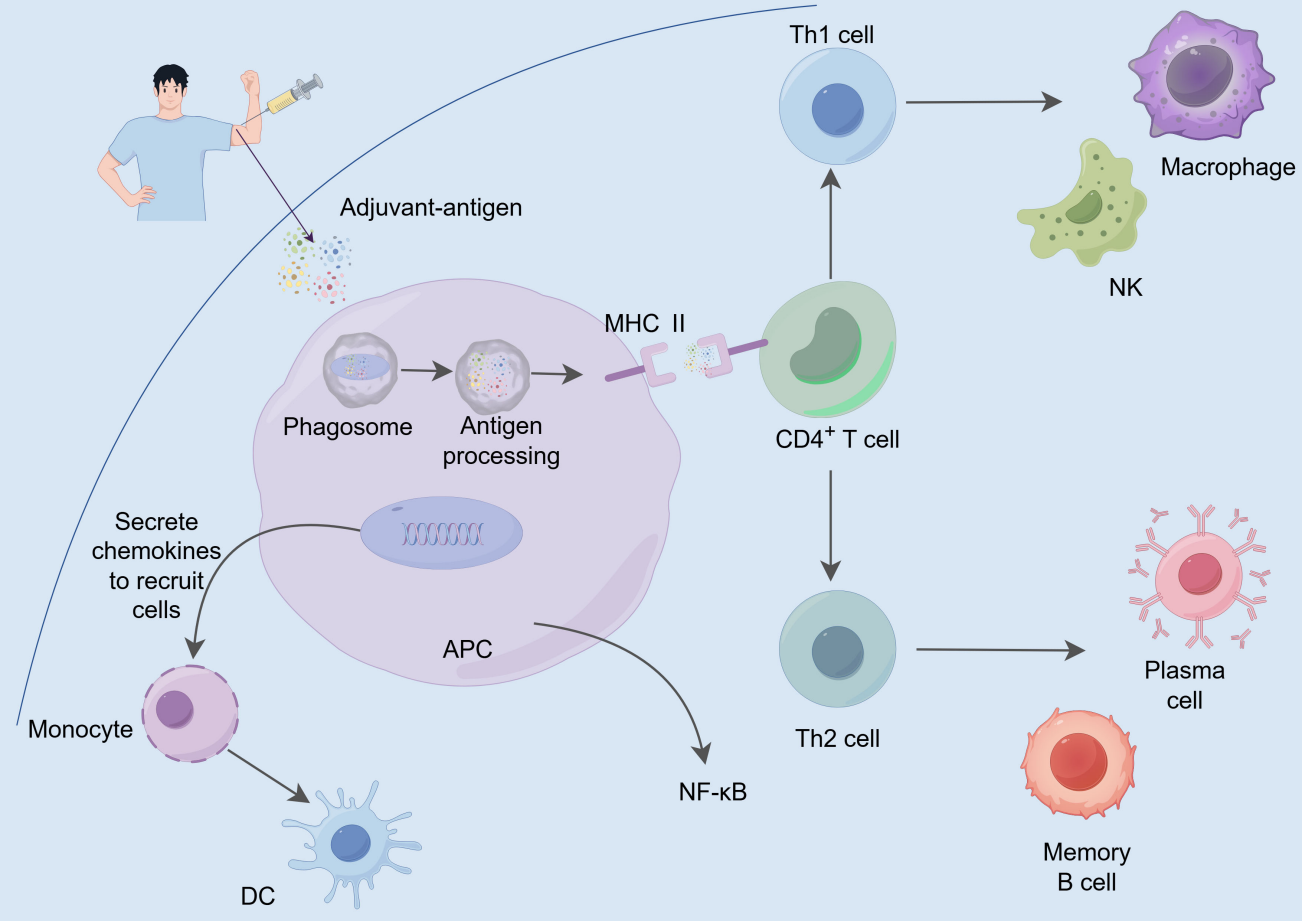
The action mechanism of emulsion adjuvants. The adjuvant-antigen complex is taken up and processed by APCs, where it is recognized by MHC II molecules and presented to CD4^+^ T cells, initiating a specific immune response. Activated Th1 cells promote the activation of macrophages and NK cells, thereby enhancing cell-mediated immunity. Meanwhile, Th2 cells promote the differentiation of B cells into plasma cells and memory B cells, boosting antibody production and enhancing humoral immunity. Furthermore, adjuvants activate signaling pathways such as NF-κB, stimulating APCs to secrete chemokines, which attract additional immune cells (e.g., monocytes and DCs) to the site of local immune responses, further strengthening the intensity and persistence of the immune reaction.

MF59 is an oil-in-water emulsion primarily composed of 4.3% squalene, 0.5% Polysorbate 80 (Tween 80), and 0.5% Sorbitan trioleate (Span 85) (39). MF59 emulsion has dual functions of antigen delivery and immune stimulation. When used as an emulsion delivery system, MF59 slowly releasing antigens in the lymph nodes, which enhances the efficiency of antigen presentation. Meanwhile, it also reduces direct contact between antigens and the immune system, thereby improving the immune response (37, 40). This slow-release effect allows more antigen signals to be presented on the surface of APCs, leading to a stronger specific immune response (**Figure 3**). Additionally, MF59 acts as an immune stimulant that activates the immune system to enhance the immune response through various mechanisms. MF59 targets specific PRRs and induces the production of endogenous danger signals, thereby activating innate immune cells (41). The use of MF59 in muscle activates innate immune cells, such as macrophages and DCs, promoting the production of various chemokines, including CCL2, CCL4, CCL5, and CXCL8 (42, 43), which play crucial roles in recruiting additional innate immune cells to the injection site. These chemokines attract more innate immune cells to the injection site, thereby enhancing the local immune response. Additionally, they promote the migration of these cells to draining lymph nodes, activating B cells and T cells. However, MF59 does not engage NLRP3, but instead requires MyD88 to enhance bactericidal antibody-based responses (44). MF59 does not activate TLR-dependent signaling pathways in DCs *in vitro*; therefore, its immune-enhancing effects may rely on MyD88-mediated. In short, vaccination with MF59 adjuvant leads to mixed Th1/Th2 biased cellular response *in vivo* (45). Currently, MF59 is widely used in various human vaccines, demonstrating good safety and efficacy in practical applications, such as influenza and pandemic vaccines (46).

##### 2.2.2.2 AS03

AS03 is an oil-in-water emulsion adjuvant composed of the surfactant polysorbate 80, squalene and DL-α-tocopherol (47). Similar to MF59, AS03 also possesses a dual mechanism of action. Firstly, it acts as an antigen delivery system, enhancing the presentation of antigen signals on the surface of APCs through a slow-release mechanism. Secondly, AS03 exhibits immunostimulatory effects by activating the NLRP3 pathway (independently of ASC activation) and the TLR pathway (independently of MyD88 activation) (7). The α-tocopherol in AS03, as an immunostimulant, exerts its immunomodulatory effects by enhancing the secretion of chemokines and cytokines such as CCL2, CCL3, IL-6, and CXCL1, promoting antigen uptake by APCs, and increasing the recruitment of granulocytes to the draining lymph nodes (48). AS03 generally induces Th2-biased immune responses and weakly affects Th1 responses (45). AS03 was licensed by the EU during the 2009 H1N1 pandemic for use in the flu vaccine (Pandemrix™, GSK) and received FDA approval in 2013 for the H5N1 avian flu vaccine, demonstrating excellent safety, immunogenicity, and immune responsiveness (49). AS03 is primarily utilized in various influenza vaccines, significantly enhancing antibody levels and prolonging their persistence by bolstering the immune response mechanisms (50).

### 2.3 Particles

#### 2.3.1 Virus-like particles

Virus-like particles (VLPs) are nanoscale polymer particles with a regular shape (51), formed by the self-assembly of viral capsid proteins. As delivery systems, advantages of VLPs include specific targeted and effective host-cell penetration, biocompatibility, and degradability (52). The diameter of VLPs typically ranges from 10 to 200 nm, enabling them to efficiently enter lymphatic vessels and target lymph nodes for uptake by specific cells (53). VLPs can induce the activation and proliferation of B cells, promoting class switch recombination (the process of changing antibody isotype) and somatic hypermutation (the enhancement of antibody affinity) (54). Additionally, VLPs can bind to and activate DCs, which capture them and trigger T cell immune responses (55). They can also increase CD8^+^ T cell activity and induced antibody responses (51). Therefore, VLPs can induce a broad range of humoral immunity (such as the production of neutralizing antibodies) and cellular immunity (including the activation of specific CD4^+^ T helper cells (Th) and cytotoxic CD8^+^ T cells), working together to enhance overall immune protection (56). Moreover, due to their highly ordered and repetitive spatial structure, they can show multivalent antigenic epitopes on their surface, thereby effectively cross-link B cell receptors (BCRs) and inducing a robust humoral immune response even in the absence of Th (19, 57). For example, the constructed porcine epidemic diarrhea virus-like particle (PED-VLP) could induce high levels of IgA and IgG and further elicited immune response skewing towards a Th2 type after immunization in mice (58).

#### 2.3.2 Virosomes

Virosomes are a vaccine platform that closely resembles the structure of natural viruses, composed of envelope proteins derived from recombinant influenza viruses, which assemble into VLPs. These VLPs do not contain viral genetic material, yet their envelopes include hemagglutinin (HA), neuraminidase (NA), and phospholipids (such as phosphatidylethanolamine and phosphatidylcholine), effectively mimicking the appearance and structure of natural viruses (22). As an effective antigen delivery system, virosomes can induce strong humoral and cellular immunity, comparable to natural infection and other potent adjuvants (59). It can also efficiently transfer antigens into the cytosol of APCs, thereby facilitating antigen processing and presentation, which in turn induces a CTL immune response (60).

The significant advantage of virosomes delivery systems lies in their ability to effectively adsorb antigens onto both the surface and internal spaces of the virosomes through hydrophobic interactions, which enhance stability. These hydrophobic interactions improve the binding stability of the antigens to the virosomes, thereby significantly enhancing the immune response. Moreover, during vaccine preparation, virosomes offer greater advantages over VLPs due to their structural flexibility and ability to effectively deliver and present antigens. This is because the protein structural characteristics of VLPs impose limitations on their mobility and self-assembly, while the more flexible structure of virosomes allows for more effective participation in antigen delivery and presentation. Furthermore, the adsorption of antigens onto the fluid phospholipid bilayer surface of virosomes can enhance the interaction between antigens and host cell receptors, promoting immune activation. The fluidity and structural characteristics of the phospholipid bilayer facilitate more effective binding of antigens to receptors, thereby promoting antigen delivery and immune activation. The FDA has approved viral particles as nanocarriers for human use, primarily due to their demonstrated high tolerability and safety in multiple studies, including applications in vaccine and therapeutic development (61–63). These approvals encompass the development of vaccines and therapeutic agents, indicating that virosomes hold significant promise for a wide range of clinical applications.

### 2.4 Mannan-based adjuvants

Mannan-based adjuvants are polysaccharide compounds composed of linearly linked mannose residues (e.g., β-(1→4) or β-(1→2) configurations) derived from natural sources such as fungi, yeast, and bacterial cell walls (64, 65). These adjuvants exert immunomodulatory effects by binding to C-type lectin receptors (e.g., mannose receptor and DC-SIGN) on antigen-presenting cells, promoting dendritic cell maturation and skewing immune responses toward tolerance or Th1/regulatory T cell (Treg) differentiation (66, 67). Preclinical studies demonstrate that mannan-allergoid conjugates enhance antigen uptake by monocytes and dendritic cells, drive IgG2a/IgG4 antibody production, and suppress IgE-mediated hypersensitivity in murine models (68). Clinically, subcutaneous or sublingual administration of mannan-conjugated allergoids significantly improved allergic symptom scores, reduced medication use, and induced allergen-specific IgG4 antibodies in phase II/III trials (69). Recent innovations include covalent linkage of mannan derivatives (e.g., oxidized mannan) to antigens for targeted delivery, as exemplified in vaccines against Leishmania and *Candida albicans*, where they enhance both humoral and cellular immunity (70). The capacities of mannan-based adjuvants to modulate adaptive immunity favorable safety them as promising candidates for next-generation allergen-specific immunotherapy and infectious disease vaccines (68).

## 3 Immunostimulants

**Figure 4:**
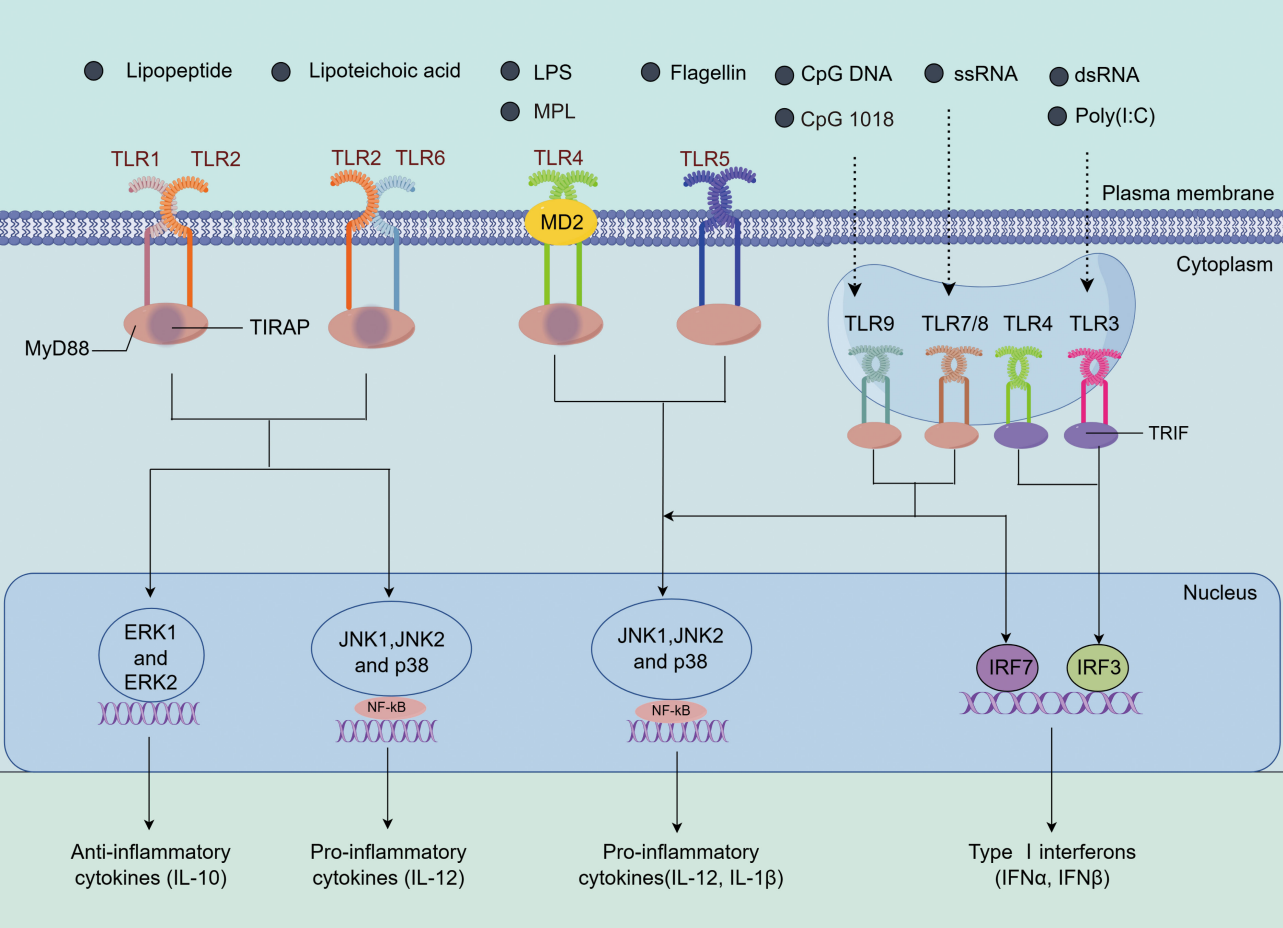
The immune activation mechanisms by TLR agonists. TLR2 can form heterodimers with TLR1 or TLR6 (TLR1/2 or TLR2/6), recognizing lipopeptides and lipoteichoic acid, among other ligands. Upon activation, TLR2 recruits the adaptor protein MyD88, initiating downstream signaling pathways, including NF-κB and the MAPK family (ERK, JNK, p38), which leads to the induction of IL-12 and IL-10 expression (72, 73). TLR4 recognizes LPS and forms the TLR4/MD2/LPS complex with the bridging protein myeloid differentiation factor 2 (MD2). It activates the MyD88-dependent pathway, which in turn activates JNK, ERK1/2, p38, and the transcription factor NF-κB, inducing the expression of IL-1β and IL-12. Additionally, TLR4 also signals through the TRIF-dependent pathway, activating interferon regulatory factor 3 (IRF3) to promote the production of type I interferons (74, 75). TLR5 activates upon recognizing flagellin and similarly induces inflammation via the MyD88-dependent pathway (76). TLR7/8 and TLR9, located in endosomes, recognize ssRNA and CpG DNA, respectively. Through their TIR domains, they recruit MyD88, activating the NF-κB and MAPK (such as JNK and p38) pathways, leading to the expression of pro-inflammatory cytokines (such as IL-1β and IL-12). Moreover, TLR7/8 and TLR9 also activate interferon regulatory factor 7 (IRF7) through TRAF3, promoting the production of type I interferons (77–79). TLR3 recognizes dsRNA and signals through TRIF, recruiting IRF3 to induce the production of type I interferons. These TLRs play important roles in immune responses through their specific signaling pathways (80).

Immunostimulants are a class of important danger signal molecules that promote the maturation and activation of APCs by specifically targeting receptors on these cells. Different types of immunostimulants signal through various PRRs, triggering the secretion of different cytokines. These cytokines play a crucial role in adaptive immune responses, determining the nature and intensity of the immune reaction. Based on their ligand recognition and structural characteristics, PRRs can be classified into several major groups: TLRs, C-type lectin receptors, nucleotide-binding oligomerization domain (NOD)-like receptors (NLRs), retinoic acid-inducible gene I (RIG-I)-like receptors (RLRs), and absent in melanoma-2 (AIM2)-like receptors (ALRs) (6, 71). Among these, TLRs are the most extensively studied and well-characterized PRRs. Compared to other PRRs, TLRs exhibit a broader ligand recognition spectrum. TLR1, TLR2, TLR5, and TLR6 are expressed on the cell surface and recognize external pathogens, while TLR3, TLR7, TLR8, and TLR9 are expressed in endosomes, primarily recognizing viruses and endogenous pathogens (7). TLR4 is expressed both on the cell surface and in endosomes (26). (**Figure 4**).

### 3.1 TLR3 Agonists

Toll-like receptor 3 (TLR3) is an endosomal receptor that detects viral dsRNA (81). Poly(I:C), a synthetic double-stranded RNA, mimics the characteristics of viral RNA and activates TLR3, thereby initiating an immune response (82). The interaction between TLR3 and Poly(I:C) promotes the production of type I interferons (IFNs), which are crucial for effectively activating conventional dendritic cells (cDCs) and further stimulating CD8^+^ T cell responses (83, 84). Type I IFNs enhance the antigen-presenting capabilities of dendritic cells, thereby boosting the activation and functionality of CD8^+^ T cells. Poly(I:C) either activate RIG-I and melanoma differentiation-associated protein 5 (MDA5), both of which recognize double-stranded RNA and initiate a series of immune responses, including the production of interferons and the release of cytokines. Therefore, the immunostimulatory effects of Poly(I:C) are not only attributed to the activation of TLR3 but also involve the collaborative actions of multiple receptors, including RIG-I and MDA5, which enhance the overall immune response (81, 85).

In addition to Poly(I:C), other TLR3 agonists include Poly(I:C12 U) (Ampligen, Rintatolimod), Poly(ICLC) (Hiltonol), and PIKA, each with unique properties and applications. Poly(I:C12 U) reduces the toxicity of Poly(I:C) by introducing mismatches between uridine and guanosine residues, which is important for enhancing safety in clinical applications (86, 87). Poly(I:C12 U) has been explored in early clinical studies as an adjuvant for influenza vaccines, anti-HIV therapeutics, and cancer vaccines, highlighting its potential across various applications (88, 89).

Poly(ICLC) is a complex formed from carboxymethylcellulose and poly-L-lysine, with similar structure to Poly(I:C), and effectively stimulates IFN production (90) while exhibiting high resistance to serum nucleases, ensuring stability and a sustained immune response *in vivo.* It can also induce the expression of genes related to innate immune pathways, including inflammasomes and the complement system, mimicking the immune responses elicited by live viral vaccines (91). The evidence supports that Poly(ICLC) has the potential to enhance immune responses as a vaccine adjuvant in various infectious diseases, including malignant malaria, HIV, and cancer (92–94). Additionally, when administered intranasally in combination with chimeric antibodies containing HIV-p24 protein, it effectively induces gastrointestinal immune responses, enhancing protection against HIV (95).

### 3.2 TLR4 Agonists

Toll-like receptor 4 (TLR4) is a key component of the innate immune system responsible for the recognition of pathogens. It activates immune responses upon binding to monophosphoryl lipid A (MPL), a low-toxicity derivative of lipopolysaccharide (LPS) obtained by dephosphorylation of LPS from Gram-negative bacteria, such as Salmonella R595. The toxicity of MPL is approximately 0.1% of that of native LPS (96). MPL preferentially activates the TRIF signaling pathway that triggers different cytokine production when compared to LPS that activates MyD88 and produces high amounts of TNF*α* (97). Specifically, MPL induces the production of interleukin-12p40 (IL-12p40), CXCL10 and TNF, which are crucial for promoting Th1 immune responses and enhancing the immune reaction against pathogens (98). A subcutaneous four-injection immunotherapy for allergic rhinitis (Pollinex Quattro), comprising specific allergoids and MPL, has demonstrated clinical efficacy and good tolerability in children (99). In addition to their use in licensed vaccines against HPV, HBV, malaria, and herpes zoster, MPL-containing adjuvants have been employed in clinically investigated vaccines for tuberculosis, *Clostridium difficile*, HIV, herpes simplex virus, Norwalk virus, and respiratory syncytial virus (RSV) (6, 31, 100).

### 3.3 TLR5 Agonists

Toll-like receptor 5 (TLR5) is a receptor that recognizes bacterial flagellin, primarily expressed in immune cells such as macrophages, dendritic cells, and epithelial cells, playing a crucial role in initiating immune responses. Upon binding with its ligand, TLR5 activates downstream inflammatory signaling pathways, leading to the release of various inflammatory mediators, including TNF-α, IL-1β, IL-6, and nitric oxide (26). The binding of flagellin to NAIP5 triggers the interaction of NAIP5 with NLRC4, leading to the activation of NLRC4. Upon detecting flagellin, NAIP5 and NLRC4 form a heterooligomeric inflammasome, which subsequently activates caspase-1. The activated caspase-1 then mediates the proteolytic processing of pro-IL-1β and pro-IL-18 (101). Even in populations with impaired immune function, such as HIV-positive individuals, flagellin maintains its effective adjuvant properties (102). Research indicates that flagellin can fuse with target antigens, allowing for co-delivery to the same APCs, enhancing vaccine efficacy. Influenza vaccines made from flagellin-hemagglutinin fusion proteins (such as VAX 128 and VAX 125) and flagellin-matrix proteins (like VAX 102) have completed preliminary clinical trials (6). Currently, at least three vaccines using flagellin as an adjuvant are in clinical trials, including two targeting influenza viruses and one for *Yersinia pestis* (6, 103, 104).

### 3.4 TLR7/8 Agonists

Current research indicates that TLR7/8 agonists can induce Th1-type immune responses and exert potent immune modulation, particularly in populations with significant immunological differences (105). Moreover, the adjuvant activity of TLR7/8 agonists can be enhanced through chemical modifications such as lipidation and phosphorylation. For instance, lipidated TLR7/8 agonists can bind more effectively to aluminum adjuvants, thereby extending their retention at the injection site, reducing systemic side effects, and boosting immune responses (106). Ligands combining with TLR7/8 can induce high levels of IFN, IL-12, tumor necrosis factor-alpha (TNF-α), and interleukin-1β (IL-1β), all of which play key roles in enhancing Th1 immune responses. Moreover, TLR7/8 and TLR9 agonists are unique in their ability to simultaneously activate and promote the clonal expansion of cDCs and plasmacytoid dendritic cells (pDCs), which are essential for enhancing immune responses. TLR7/8 agonists include imiquimod (R837) and resiquimod (R848), which are synthetic small molecules of imidazoquinoline with significant immunostimulatory effects (107). Recent study shows that imidazoquinoline can mimic single-stranded RNA (ssRNA) recognized by TLR7/8 in endosomes, triggering immune responses through the MyD88 signaling pathway (108).

Imiquimod (Aldara) has been approved for the treatment of actinic keratosis, basal cell carcinoma (108) and genital warts caused by HPV (109), demonstrating its broad clinical application through its immune-modulating properties. Additionally, resiquimod has been tested in clinical trials for the treatment of lesions caused by human herpesvirus (HSV) (110, 111), with results indicating its effectiveness in achieving significant therapeutic outcomes. When these molecules are directly conjugated with aluminum adjuvants, they can significantly enhance vaccine immunogenicity by improving antigen presentation and immune responses (112). Previous studies have shown that the direct conjugation of imidazoquinoline with HIV Gag protein or inactivated influenza virus significantly enhances Th1 immune responses and increases the quantity of antigen-specific T cells (108, 113). Additionally, conjugating imidazoquinoline with synthetic polymer scaffolds, lipid polymer amphiphiles, polyethylene glycol (PEG), nanogels, and aluminum adjuvants have significantly improved delivery efficiency and maturation of dendritic cells and antigen-specific T cells (114). Previous research indicates that combining imidazoquinoline with other TLR agonists such as MPL (TLR4) and MPL + CpG ODN (TLR4 and TLR9) can significantly enhance innate immune responses, leading to increased production of antigen-specific neutralizing antibodies and improved Th1 responses (115–117). Bharat Biotech used a gelatin-adsorbed TLR7/8 agonist (Algel-IMDG) as an adjuvant in its whole virus particle inactivated SARS-CoV-2 vaccine BBV152 (COVAXIN) (118–120). The BBV152 vaccine is able to enhance both humoral and Th1-skewed cellular immune responses after immunization by optimizing the inactivated vaccine formulation (121). These innovative studies highlight the exceptional potential of TLR7/8 agonists as adjuvants, demonstrating significant advantages in enhancing immune responses and vaccine efficacy.

### 3.5 TLR9 Agonists

Toll-like receptor 9 (TLR9) is localized intracellularly in endosomal membranes and detects single-stranded unmethylated CpG oligodeoxynucleotides (CpG-ODN) of bacterial and viral DNA (122). CpG ODN is a synthetic, short, single-stranded DNA molecule that can be flexibly synthesized. It can mimic bacterial CpG DNA, achieving a highly similar immunostimulatory effect. By directly activating B cells and plasmacytoid dendritic cells (pDCs), CpG ODNs induce both innate and adaptive immune responses. As a result, they serve as potent TLR9 agonists (123). TLR9 transmits signals via MyD88, IRAK, and TRAF-6 (99), ultimately leading to the upregulation of co-stimulatory molecules (CD40, CD80, and CD86) as well as pro-inflammatory cytokines (IL-6, IL-12, IL-18, and TNF-α) (55). To date, three classes of CpG-ODN ligands (A-C) have been developed, but only Class B CpG-ODN has been used as an adjuvant in clinical trials (123). CpG-B ODN primarily localizes in endosomes, triggering the maturation of pDCs, thereby enhancing immune responses (77). Additionally, CpG-B ODN can bind directly to B cells, stimulating their proliferation and enhancing antibody production. Also, the immunogenicity of aluminum-based vaccines can be enhanced by CpG ODN, including those for hepatitis B, anthrax, and influenza (124). Meanwhile, it enables to vaccines to exert its immune effect *in vivo via* intramuscular, subcutaneous, oral, and intranasal routes (125). The licensed CpG 1018 is an oligonucleotide with high chemical stability and potent adjuvant properties, inducing Th1-type immune responses and used as an adjuvant in the hepatitis B vaccine Heplisav-B (10). Overall, these studies indicate that CpG ODN, particularly Class B ligands and CpG 1018, hold significant potential for enhancing immune responses and vaccine efficacy, providing crucial scientific evidence for the development and optimization of future vaccine adjuvants.

## 4 Combination of adjuvants

Recently, using various combinations of adjuvants to activate different signaling pathways in order to optimize vaccine immune responses has proven to be a promising approach (2). These observations stem from studies investigating the activation of different PRRs induced by effective live attenuated vaccines, such as the yellow fever vaccine (126). Based on these findings, using different TLR agonists to activate distinct signaling pathways, such as MyD88 and TRIF, is a good strategy (127) Previous studies tested various combinations of TLR agonists in human peripheral blood mononuclear cells (PBMCs) and assessed their impact on cytokine and chemokine production (127). The combination of TLR7 and TLR9 agonists induces the production of IFN-I; TLR4 combined with TLR7/8 synergistically upregulates levels of IFN-γ and IL-2; while TLR2 and TLR7/8 together significantly enhance levels of cytokines such as IFN-γ (55). The combined use of MF59 and Carbopol-971 P significantly increased the specific antibody titers against HIV (128), indicating a positive role of this adjuvant combination in enhancing immune responses. However, not all combinations enhance the strength of the immune response; for instance, immunizing mice with recombinant HIV gp 140 in combination with MPL and aluminum adjuvants or Muramyl dipeptide (MDP) enhance the magnitude and quality of humoral immune responses, though the effects vary depending on the combinations used. When mixtures include MDP with poly(I:C) or resiquimod, there is no significant effect on antibody titers, but notable differences in the distribution of IgG subclasses are observed (129).

Another study indicated that using nanoparticles containing both antigen and TLR4 and TLR7 ligands significantly enhances the production of antigen-specific neutralizing antibodies compared to the use of nanoparticles containing only the antigen and a single TLR ligand (130). The effects of different TLR ligand combinations on the activation of DCs were also evaluated. It is suggested that the combination of TLR3 and TLR4 agonists with TLR8 agonists effectively synergized, inducing higher levels of IL-12 and IL-23 than the optimal concentrations of the agonists used individually in human dendritic cell (55). This synergy enhances the polarization capacity of Th1-type immune responses and prolongs their duration. This optimization strategy, by precisely combining different TLR agonists with other adjuvants, can more effectively enhance vaccine immune responses, thereby improving vaccine efficacy.

The adjuvant systems developed by GlaxoSmithKline over the past thirty years are designed based on combinations of classic adjuvant molecules, such as aluminum adjuvants, emulsions, and liposomes, used in conjunction with immunostimulatory molecules (e.g., TLR ligands) to achieve optimal adjuvant effects while ensuring good tolerability (5). Currently, GlaxoSmithKline has developed several adjuvant systems, including AS01, AS02, AS03, and AS04, with AS01, AS03, and AS04 widely applied in commercial vaccines, while some systems remain in clinical trial phases. The continued development and evaluation of these adjuvant systems may provide new avenues for enhancing vaccine efficacy and safety (131).

### 4.1 AS01

AS01, as a liposome-based adjuvant, contains monophosphoryl lipid A and a saponin known as QS-21, which act synergistically to induce strong antibody and helper T cell responses (132). It is included in the approved varicella-zoster virus vaccine Shingrix, which was specifically designed for individuals aged 50 and has demonstrated an efficacy was up to 97.2% (133). QS-21 is a purified component (component 21) extracted from the bark of Quillaja Saponaria Molina, containing water-soluble triterpene saponins that play a crucial role in stimulating the immune system. In AS01, MPL and QS-21 are formulated with cholesterol in liposomes, where cholesterol aids in the binding of QS-21 to the liposomes and reduces its potential reactivity. MPL activates the innate immune system by binding to TLR4, primarily functioning through a TRIF-dependent signaling pathway (5).

Furthermore, mouse model studies have shown that QS-21 can activate caspase-1 in subcapsular sinus macrophages (SSM) within draining lymph nodes, which plays a critical role in regulating immune responses (134). When formulated in liposomes, QS-21 enters cells through a cholesterol-dependent endocytosis mechanism, inducing lysosomal destabilization and further activating the tyrosine kinase SYK (135). In summary, AS01 combines two established adjuvant molecules within a novel delivery system (liposomes) to produce synergistic innate immune effects, leading to a significantly superior adaptive immune response compared to the individual components used alone. It can be attributed to the two combined effects. On the one hand, an innate cell exposed to MPL and QS-21 enable to occur complex signal integration due to the common signaling pathways. On the other hand, MPL and QS-21 enable the creation of a network of innate effector functions by activating different cell types. Under the above two mechanisms, the signal produced by innate cells can be amplificated, thereby resulting in an improved response to vaccine antigens (136).

### 4.2 AS02

The adjuvant AS02, as an oil in water emulsion composed of MPL and QS-21 (4), has been widely used in tumor immunotherapy, tuberculosis, hepatitis B, and malaria (17). Under the action of synergy between QS-21 and MPL, high levels of IFN-γ is produced, which is a typical cytokine for CD4-type cellular response, further stimulating both humoral and cellular immune responses (16). In addition, intramuscular injection offers greater advantages over subcutaneous injection in enhancing the immunogenicity and safety of the RSV-F vaccine when combined with the AS02 adjuvant following enhanced immunization, particularly in comparison to the MF59 adjuvant (137).

In the two adjuvant systems AS01 and AS02, they respectively employ different mechanisms of action to stimulate the immune response (5, 138). AS01 combines MPL and QS-21 via a liposomal delivery system, triggering a strong Th1-biased immune response that has been shown to be effective in several vaccines, such as Shingrix, RTS,S/AS01E, and RSVPreF3-AS01E (136, 139). In contrast, AS02, formulated with an oil-in-water emulsion, promoted a humoral immune response to Th2 migration by extending antigen retention at the injection site, showing potential against certain antibody-dependent diseases. The two adjuvant systems, both developed by GSK, differ in their mechanisms due to differences in their components and immune activation pathways. While AS01 has been successful in licensed vaccines due to its liposomal delivery and dual-pathway synergies, and AS02 is still in the experimental phase, early trial results have highlighted its potential value in the treatment of diseases such as malaria and tuberculosis (140).

### 4.3 AS04

AS04 contains 3-O-desacyl-4′-monophosphoryl lipid A (MPL), a detoxified form of LPS derived from *Salmonella*. MPL is adsorbed onto aluminum adjuvants, enhancing the immune response through its immunostimulatory properties. Compared to adjuvants that contain only aluminum adjuvants, AS04 significantly enhances the durability and efficacy of the immune response in HPV vaccines (141).

Research has shown that when AS04 is used as an adjuvant, cytokines such as IL-6 and TNF-α are rapidly produced at the injection site and in the draining lymph nodes within 3–6 hours, recruiting various immune cells, such studies demonstrate that MPL is a key component mediating the early immune response in vaccines by activating innate immune pathways (16). Although the combination of aluminum adjuvants and MPL do not show a synergistic effect, aluminum adjuvants can prolong the duration of the cellular response initiated by MPL at the vaccination site. Thus, the results indicate that the AS04 adjuvant induces an innate immune response primarily through the activation of TLR4 (142).

Compared to the use of aluminum hydroxide alone, the AS04 adjuvant significantly increased IFN-γ levels after binding with HPV-16 and HPV-18 VLP antigens, indicating a stronger Th1-skewed response since IFN-γ is a key marker of this type of immune response. These results suggest that AS04 is more effective in inducing the proliferation and differentiation of CD4^+^ T cells, further promoting a Th1-skewed immune response (142). The HBV vaccine containing AS04 significantly enhances the innate immune response in humans, which is crucial for initiating a robust adaptive immune response (143). Compared to the HBV vaccine (Engerix-B) with aluminum adjuvant, the HBV vaccine (FENDrix) with AS04 adjuvant triggers a stronger immune response in both humoral and cellular immunity. In the immune serum with AS04 adjuvant, IL-6 and C-reactive protein levels are significantly elevated, indicating a stronger inflammatory response, which is generally associated with enhanced immune system activation. Additionally, AS04 adjuvant induces higher levels of hepatitis B virus surface antigen (HBsAg)-specific T cells and antibodies, demonstrating its clear advantage in activating specific immune responses and enhancing immune memory (143). Furthermore, compared to the aluminum adjuvant vaccine, the HBV and HPV vaccines formulated with AS04 adjuvant induce a stronger humoral immune response, highlighting the crucial role of the TLR4 agonist MPL adjuvant in enhancing immune responses (16, 144, 145).

## 5 Conclusions and prospects

Adjuvants play a crucial role in vaccine development by significantly enhancing the immune response elicited by vaccines, thereby improving their overall efficacy. With significant advancements in adjuvant research over the past two decades, scientists can now select the most suitable adjuvants from classic options (such as aluminum adjuvants) and immunostimulants (such as TLR agonists) or their combinations to enhance vaccine efficacy. This article firstly summarizes the potential benefits and key characteristics of adjuvants, then categorizes them into delivery systems and immunostimulants based on their mechanisms of action, providing detailed explanations of the mechanisms and applications of them. Finally, we summarize the adjuvant systems formed by the combination of classic adjuvant molecules and immunostimulatory agents, exploring the practical applications of these innovative combinations in vaccine design. These systems offer diverse options that aid in optimizing vaccine design, exploring new adjuvant formulations, and facilitating the effective development of vaccines against diseases. There are still several questions that need to be investigated in the future study.

Selecting the appropriate adjuvant is crucial in the development of new vaccines, as adjuvants significantly enhance the immune response elicited by vaccines, thereby improving their overall efficacy. Since no single adjuvant is suitable for all types of antigens, it is essential to select the most appropriate adjuvant based on the immune system’s specific responses to different vaccines. For example, to effectively control intestinal pathogens, oral vaccines must overcome degradation and immune tolerance issues that antigens may encounter in the gastrointestinal tract, presenting significant challenges for vaccine design and development (146). To address these challenges, it is necessary to utilize biodegradable micro- or nanoparticles that can withstand low pH environments and target antigens to M cells in the intestine, thereby enhancing antigen stability and immune responses. U-Omp 19, a bacterial protease inhibitor extracted from *Brucella abortus*, serves as an adjuvant for oral subunit vaccines by inhibiting proteases in the stomach and intestine, delaying antigen digestion in lysosomes, thus enhancing antigen presentation efficiency and promoting the recruitment of immune cells in the gastrointestinal mucosa (147). Additionally, selecting the appropriate adjuvant must take into account individual factors such as the recipient’s age, medical history, and genetics, as these factors can significantly influence the immune system’s response to vaccines (148–151). The cost of production varies greatly among different types of adjuvants. For example, some adjuvants aluminum salt-based adjuvants are relatively inexpensive and widely available, while some newer adjuvants, such as water-in-oil emulsions or genetically engineered adjuvants are usually expensive to manufacture. Therefore, the safety and cost of the adjuvant must be comprehensively considered, as these factors not only affect the practical application of the adjuvant but also potentially influence the overall efficacy of the vaccine.

Furthermore, in practical applications, adjuvants may present various adverse effects. For example, while immunostimulants can effectively induce immune responses, they may also lead to adverse reactions, such as autoimmune diseases (7, 152, 153). Adjuvants not only enhance the immune response to antigens, especially certain adjuvants that can boost specific CTL responses, but they may also cause short-term side effects (such as fever, headache, or flu-like symptoms) (125) and serious side effects (such as sterile abscesses, granuloma formation, and local swelling at the injection site) (4, 154). Moreover, many adjuvants are limited in clinical applications due to their complex production processes, poor stability, and potential to induce immune tolerance. An ideal adjuvant should possess a broad safety profile, be easy to produce and use, effectively activate both humoral and cellular immune responses, and avoid adverse reactions. Therefore, in-depth research on the mechanisms of adjuvants and a comprehensive understanding of their effects on the immune system are essential. Additionally, structure-activity relationship analyses of immune adjuvants are crucial for enhancing their efficacy and safety.

A number of studies have indicated that synergistic combinations of multiple adjuvants can significantly enhance vaccine efficacy, offering advantages such as reduced dosage, lower side effects, decreased toxicity, and cost-effectiveness. For example, the two key components in AS01, MPL and QS-21, play essential roles in enhancing immune responses. MPL, a TLR4 agonist, induces T cells to produce IFN-γ and promotes antibody isotype switching to IgG2a/c in mouse models. QS-21, on the other hand, is capable of inducing neutralizing antibodies and helper T cell responses (132). AS01 relies on the synergistic effects of MPL and QS-21 to achieve optimal adjuvant efficacy. When used in conjunction with malaria antigens, AS01 can induce a rapid and transient innate immune response at the injection site and in the draining lymph nodes. It activates immune cells, including antigen-presenting cells, and generates antibody titers that are 20 times higher than those from natural exposure (155). The combination of multiple adjuvants in AS01 not only significantly enhances the immune efficacy of vaccines but also demonstrates the crucial role of synergistic effects in boosting immune responses, providing new ideas and strategies for future vaccine development.

With advancements in science and technology, particularly in the widespread use of subunit and recombinant vaccines, the demand for effective adjuvants is expected to grow. Through the development of interdisciplinary, continuous innovation and improvement, future adjuvants will be safer and more effective. In summary, selecting and developing appropriate adjuvants is crucial for achieving more efficient vaccine design, and ongoing research in this field will have a profound impact on public health.
